# Dignity and the provision of care and support in ‘old age homes’ in Tamil Nadu, India: a qualitative study

**DOI:** 10.1186/s12877-022-03272-4

**Published:** 2022-07-14

**Authors:** Vanessa Burholt, E. Zoe Shoemark, R. Maruthakutti, Aabha Chaudhary, Carol Maddock

**Affiliations:** 1 School of Nursing/School of Population Health, Faculty of Medical and Health Sciences, University of Auckland, Room 235B, Level 2, Building 505, 85 Park Road, Grafton, The University of Auckland Private Bag, 92019 Auckland, New Zealand; 2 Centre for Innovative Ageing, Faculty of Medicine, Health and Life Science, Swansea University, Swansea, Wales UK; 3 Department of Sociology, Manonmanian Sundaranar University, Tirunelveli, Tamil Nadu India; 4 Anugraha, Swabhiman Parisar, Kasturba Nagar, Shahdara, Delhi India

**Keywords:** Residential care, Respect, Cross-sectional studies, Privacy, India, Long-term care, Leisure activities, Reference standards, Delivery of health care

## Abstract

**Background:**

In 2016, Tamil Nadu was the first state in India to develop a set of Minimum Standards for old age homes. The Minimum Standards stipulate that that residents’ dignity and privacy should be respected. However, the concept of dignity is undefined in the Minimum Standards. To date, there has been very little research within old age homes exploring the dignity of residents. This study draws on the concepts of (i) status dignity and (ii) central human functional capabilities, to explore whether old age homes uphold the dignity of residents.

**Objectives:**

The study was designed to obtain insights into human rights issues and experiences of residents, and the article addresses the research question, “to what extent do old age homes in Tamil Nadu support the central human functional capabilities of life, bodily health, bodily integrity and play, and secure dignity for older residents?”.

**Method:**

A cross-sectional qualitative exploratory study design was utilised. Between January and May 2018 face-to-face interviews were conducted using a semi-structured topic guide with 30 older residents and 11 staff from ten care homes located three southern districts in Tamil Nadu, India. Framework analysis of data was structured around four central human functional capabilities.

**Results:**

There was considerable variation in the extent to which the four central human functional capabilities life, bodily integrity, bodily health and play were met. There was evidence that Articles 3, 13, 25 and 24 of the Universal Declaration of Human Rights were contravened in both registered and unregistered facilities. Juxtaposing violations of human rights with good practice demonstrated that old age homes have the potential to protect the dignity of residents.

**Conclusion:**

The Government of India needs to strengthen old age home policies to protect residents. A new legislative framework is required to ensure that all old age homes are accountable to the State*.* Minimum Standards should include expectations for quality of care and dignity in care that meet the basic needs of residents and provide health care, personal support, and opportunities for leisure, and socializing. Standards should include staff-to-resident ratios and staff training requirements.

## Background

In recent decades India has witnessed significant improvements in public health, with increases in life expectancy and longevity alongside declines in infant mortality and fertility rates. As a result, the age structure of India’s population has changed with increases in the proportion and absolute number of older adults (60 + years) in the population. Overall, the proportion of older people has increased from 5.4% in 1950 to 9% in 2020. However there are variations in the age structure across Indian states: in 2020, around 14% of the population in Kerala were 60 + years compared to 7% in Assam [1]. Although there have been gains in increased life expectancy and healthy life expectancy in India, there have also been increases in the proportion of the older population spending more years living with a disability. This is related to the impact of infectious diseases, malnutrition, and the rapid growth in the prevalence of non-communicable diseases (e.g. diabetes, cardiovascular disease, and hypertension), with many older people requiring long-term care and support to manage their daily activities [2, 3].

There are a variety of family forms in India, however, the notion of a normative traditional mutigenerational household and extended family prevails [4]. There is a social expectation that the traditional family will uphold filial piety (respect and obligations towards parents) and familism (prioritizing family needs above all others) [5] and meet the social, instrumental, economic and emotional needs of older people [2]. Indeed, this expectation is formally constituted in law. The Maintenance and Welfare of Parents and Senior Citizens Act mandates children, grandchildren and other relatives with sufficient resources to provide support to older people who are unable to maintain themselves. In situations where support is not provided, older people can take relatives to a tribunal to obtain a maintenance order. Non-compliant relatives may be fined or imprisoned. However, this is not a common course of action because there is a lack of awareness of the Act [6]. Furthermore, older people are reluctant to pursue legal action which could bring shame on the family and criminalise family members or result in a court order requiring the older person to transgress social norms and live with relatives other than sons [4]. Additionally, not all older people have access to family care: some do not have an extended family and/or have care needs that exceed family care-giving capabilities [4]. To cater for an increasing number of older people who need extra-familial support in later life, a new ‘old age home’ sector has emerged in India. We use the official terminology ‘old age home’ throughout this article when we refer to the sector in India. We use the expression ‘inmates’ to describe residents of old age homes. We do not condone the use of this term, but use it to illustrate the widespread adoption of the English language word (and meaning) in Indian academic, policy, media and public discourse. 

The old age home sector comprises not-for-profit and private homes. The private sector caters predominantly for ‘middle class’ older people, that can afford them [7] and the charitable (not-for-profit) sector provides for older people without financial assets. The Integrated Programme for Senior Citizens provides basic amenities for older people without access to support (e.g. food, shelter and medical care) and is administered through grants at the state level that are paid directly to providers of registered old age homes and day centres [8]. Only 310 homes were funded through this scheme in 2018–2019 across all states in India [9]. There are no accurate records of the number of old age homes in India, nor of the number of residents in facilities, as homes that do not receive funding are not obliged to obtain a license, register, or to be inspected [10].

In 2016, Tamil Nadu was the first state in India to develop a set of Minimum Standards for old age homes that are delivered by not-for profit organisations [11]. These focus on physical elements of the facilities (e.g. the size of room, presence of CCTV), access to basic services (e.g. productive activities for residents, housekeeping and assistance with daily activities) and medical services. Although there are no standards relating to the quality of care, the guidance specifically notes, that *“each inmates [sic] right to dignity and privacy should be respected”* [11]. This statement is aligned to Article 1 of the Universal Declaration of Human Rights (UDHR) [12], that all human beings are born free and equal in dignity and rights. However, the concept of dignity is complex and contestable, and is undefined in the Minimum Standards.

There are two main definitions of dignity which distinguish between inherent dignity and status dignity. The Kantian notion of inherent dignity is conceived as equal moral status and personhood which is grounded in humans’ sentience, rationality and capacity for autonomy [13]. Some authors suggest that this definition excludes people who lack cognitive capacity or autonomy (e.g. older people with severe dementia) from equal respect and dignity [14, 15]. Furthermore, many argue that inherent dignity is built on metaphysics or theology concerning the moral standing of human beings in relation to their ‘gods’ versus the rights of other animals [16], while others have argued that it is concerned with the worth of the individual in relation to other people [17]. The controversy concerning the concept of inherent dignity tends to detract from the political function of the UDHR which are intended *“to protect individuals against the consequences of certain actions and omissions of their governments”* [18]. Consequently, in this article, the concept of status dignity is used to describe the relationship of residents in an old age homes to the State and the agents of the State (staff in old age homes) [19].

Valentini [19] defines status dignity as *“a status a human being possesses, comprising stringent normative demands”* (p. 865). From this theoretical perspective, the duties to ensure the dignity of citizens (and that human-rights are fulfilled) primarily falls on the State and its agents. However, in order to explore whether the state is fulfilling their primary duty requires a definition of ‘normative demands’ essential for dignity [20]. In this respect, Nussbaum [21] has posited that governing bodies should secure for all citizens a threshold of ten central human functional capabilities (CHFC). CHFC are *“opportunities that people have when, and only when, policy choices put them in a position to function effectively in a wide range of areas that are fundamental to a fully human life”* [22].

The capability approach refers to the opportunities and freedom to undertake the activities necessary for survival, to avoid or escape poverty or serious deprivation and achieve a life that is *“not so impoverished that it is not worthy of the dignity of a human being”* [23]. For example, bodily health (a CHFC) is partly underpinned by nourishment. Nourishment in turn requires resources to prepare meals (i.e. access to food products that are culturally or religiously acceptable and an energy source to cook upon) and the personal ability or external support to undertake the functions of cooking and eating. The capability approach resonates with other authors’ descriptions of the conditions necessary to support dignity in organizational and clinical settings [20, 24, 25]. All ten CHFC are relevant to supporting the dignity of residents in old age homes, however, this article focuses on four: life, bodily health, bodily integrity and play which correspond to Articles 3, 13, 25 and 24 of the UDHR (Table 1).

**Table 1:** Correspondence between four central human functional capabilities [21] and articles of the United Declaration of Human Rights [12]

Central Human Functional Capabilities	Articles of the United Declaration of Human Rights
*Life.* Living to the end of natural human life, not dying prematurely or before one's life is reduced as not worth living	*Article 3*. The right to life, liberty and security of person
*Bodily health.* Able to have good health, to be adequately nourished, and have adequate shelter	*Article 25.* The right to a standard of living adequate for health and well-being, including food, clothing, housing and medical care and necessary social services, and the right to security in the event of unemployment, sickness, disability, widowhood, or old age
*Bodily Integrity.* Able to move freely from place to place, secure against violent, sexual and domestic assault	*Article 13.* The right to freedom of movement and residence within the borders of each state
*Play.* Able to laugh and play and enjoy recreational activities	*Article 24*. The right to rest and leisure

In India, there has been very little research within old age homes. The research that has been published has tended to focus on the private sector [7]. The available evidence suggests that a majority of homes require residents to be ambulatory, continent, and cognitively able at the time of admission [7]. Whether the CHFC are supported for residents that become unable to self-care because of physical or cognitive impairment is unknown. Presently, it is unclear as to the extent to which staff in old age homes, as agents of the State, uphold the dignity of residents. To explore human rights issues and experiences of old age home residents in India, this article addresses the following research question:

*To what extent do old age homes in Tamil Nadu support the central human functional capabilities of life, bodily health, bodily integrity and play, and secure dignity for older residents?*

## Methods

### Sample location

Tamil Nadu state is situated in the south India and covers 130,060km^2^. Tamil Nadu had a population of 72 million in 2011 of which 88% were Hindu. One-tenth (*n* ≈ 7.2 million) of the population were age ≥ 60 years.

### Sampling procedures

**Fig. 1:**
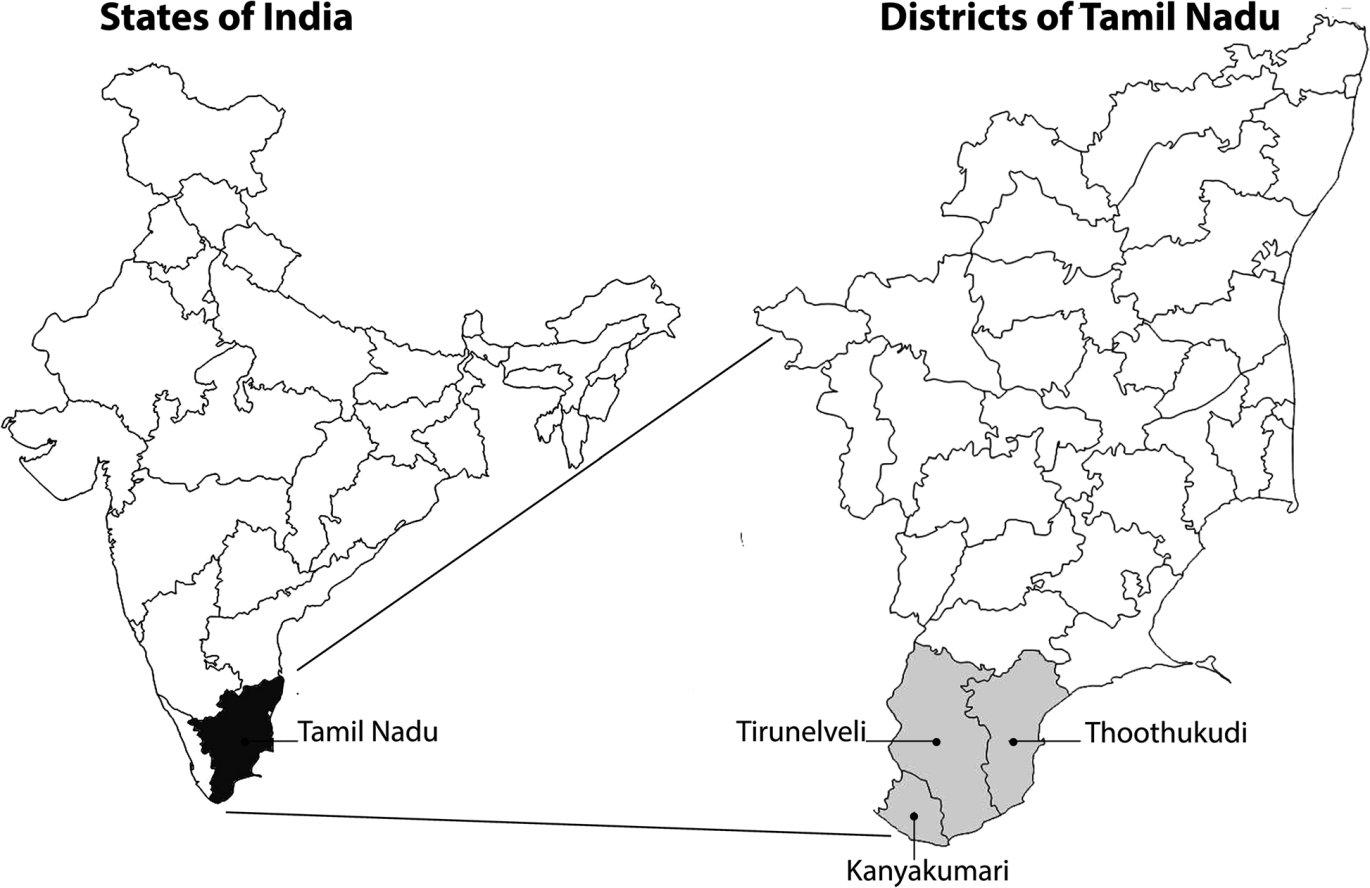
Map of the states of India showing the location of Tamil Nadu, and map of Tamil Nadu showing location of selected states

Old age homes were purposively selected from three southern districts in Tamil Nadu: Thoothukudi, Tirunelveli, and Kanyakumari (Fig. 1). Forty-three old age homes were located through a mapping exercise: 13 in Thoothukudi, 11 in Tirunelveli and 18 in Kanyakumari. The ratio of fee-paying to free old age homes in each district, and the size of the homes were used to inform our sampling strategy. Participants were randomly selected from lists of residents in 10 facilities, to obtain (as far as possible) a gender-balanced sample of 10 people in each district (Table 2).

**Table 2:** Characteristics of old age homes in sample

					Staff						
ID	Reg	Rs per month	Number of Residents	Residents in Sample	Manager	Care attendant	Cleaner	Cook	Security	Other	Staff in sample
H10	No	3,000 5,000 8,000	32	2	1	0	3	3	0	Gardener	1
H11	No	Free	110	6	1	0	0	2	0		1
H15	No	Free	25	4	1	0	1	1	0	2 servants	2
H16	No	Free	20	4	1^b^	0	0	2	0		1
H12	Yes	Free	8	2	1	2	1	1	0		1
H13	Yes	5,000 8,000	5	2	1	1	1	1	1		1
H14	Yes	Free^a^	65	4	2	17 staff across categories					1
H17	Yes	Free	11	2	1^c^	1^d^	0	1	1		2
H18	Yes	10,000 13,000	18	4	1^c^	3	1	1		Accountant	1

^a^Purports to be for destitute older people: of those interviewed, all participants either paid themselves or had relatives that paid ‘donations’

^b^Also role as care attendant

^c^Also role as nurse

^d^Also role as cleaner

### Data collection

Face to face guided interviews (17–70 min; *M* = 34 min) were conducted in Tamil with 30 residents (15 male, 15 female, age range 60–83 years) and 11 staff in old age homes, between January and May 2018 by three experienced female interviewers who were PhD scholars at Manonmanian Sundaranar University. To standardise approaches to interviewing, training was provided by the first and third author.

Interviewers explained the purpose of the study and established relationships with the residents and staff before the study commenced. All participants were interviewed in a private place where they could not be overheard or interrupted.

Semi-structured interview guides were used for residents and staff. Open-ended questions explored how residents came to be living in the old age home [described elsewhere, 4] and experiences of the old age facility. Examples of questions included: *“Tell me about your typical day”, “What is the best thing about living here?” “What is the worst thing about living here?” “If you need help here, does anybody help you?” “What do you do with your time?”* Staff were asked questions such as,*“What services are provided to residents?” “How are residents’ needs assessed, if at all?” “What happens if a resident becomes sick?”.*

The first three interviews were used to pilot the interview guide, and to check the quality of interviewing. Interviews were recorded, transcribed, and translated by a professional translator into English and anonymised. Pseudonyms are used throughout the article.

### Analysis

Framework analysis was used to analyse the data [26]. Five distinct but inter-connected phases (familiarisation; identifying a thematic framework; indexing; charting; mapping and interpretation) provided a rigorous methodological structure. Familiarisation, conceptual and cultural understanding of the interviews were clarified during team meetings (first and third author with interviewers) in Tamil Nadu. The second author created a list of a priori codes from the interview questions (e.g. support for each activity of daily living, discriminatory practices, food and nutrition, religious practices, leisure and recreation, worst and best things in the home). She applied these codes to transcripts (in NVivo 1.5.1), and while reading through the transcripts simultaneously created a new series of a posteriori codes, in an inductive thematic analysis. The first author read through the transcripts and coding to check validity. She then systematically applied a second coding index based on the capability approach: some thematic nodes became wholly subsumed in the ‘family’ nodes (e.g. ADLs were subsumed under ‘bodily health’) whereas others were relevant to more than one CHFC (e.g. worst thing in home). The first author charted the data into a framework which provided a decontextualized descriptive account of the data in relation to each CHFC.

The first author undertook the preliminary interpretation of data in three steps. First, taking an outcome-oriented approach, examples where each CHFC had/had not been secured were juxtaposed, providing an insight into the breadth of experiences of old age home residents. Second, residents’ and staff interviews were grouped by home and particular attention was paid to exceptions, contradictions and disconfirming excerpts. Third, experiences were contrasted between types of facilities (registered versus unregistered). All other authors (in India and the UK) were used as a sounding board, to check the persuasiveness of the analysis and to provide different ways of interpreting the research phenomenon [27].

## Results

### Life

‘Life’ refers to being able to live a normal life-span and not dying prematurely or before one's life is reduced as not worth living. This CHFC is closely related to bodily health. Participants referred to the ceiling of care available to them, if a resident was ill, or unable to carry out activities of daily living. In some cases, this was also referred to as the ‘worst thing’ about the old age home.

The first quote highlights the practical and ethical difficulties of deciding what ‘a life not worth living’ comprises. In this example, the resident perceived that the facility would judge that a sick older person’s life had no quality and would not be worth saving. Maalia explained what would happen if she became ill in the old age home in which she lived:

*They will put me in the back room [sick room]. You may get a bath or you may not. You will have to lie there and die… If I become sick, the Sister will pour coffee and porridge. They will do that only when you become too sick. When you are going to die, they will pour water and bid farewell. That’s all.* (Female, 65 years, widowed, H11 unregistered)

In other facilities, residents believed that if their health deteriorated to a point at which they were unable to take care of themselves they would be cared for by their children. For example, Rishi who had four daughters and lived with his wife said:

*If we become too old and unable to do things, they [daughters] will only take us back. Now we are able to do things. So we are here. If we fall sick, these people will inform them and they will come and take us with them.* (Male, 83 years, married, H14 registered)

Similarly, Hitendra said:

*What if I become sick in the last stage of my life? Luckily I joined here when I was healthy. The facilities here are good. So, I want to be here for some time... I told my son that I would go to his house in [city* > *200 miles away] if my health deteriorated.* (Male, 83, separated, H10 unregistered)

The expectations for support at the end of life maybe unrealistic, as the residents were living in the facilities because their families were unwilling or unable to provide support. If support did not materialise, then this would not be problematic for Rishi, as the old age home he lived in provided life-long support. Diya, another resident in H14 noted:

*If we are not well, the doctor will immediately attend. All medicines will be provided immediately. These three people [the doctor, the manager and the assistant manager] take such good care. One should have done punniyam [meritorious deeds in previous lives or in the past] to come here.* (Female, 77, widowed, H14 registered)

However, there was no health care, personal support, or palliative care in the facility where Hitendra resided, and the manager noted:

*The residents should take care of themselves. If they cannot take care of themselves, we cannot help. We do not have any attendant here.* (Manager, H10 unregistered)

The Tamil Nadu Minimum Standards note that *“each Old Age Home should ensure that the inmates [sic] should continue to receive care till the end of his/her life or up to natural death”* [11]. Despite, mandatory care obligations, the Standards were only enforceable in registered facilities. Consequently, with one exception where a nun provided some personal support (see section on bodily health below), there were no care attendants to support residents in the unregistered facilities (Table 2).

### Bodily health

This theme incorporated examples of supporting residents’ bodily health through medical and personal care (i.e. support for activities of daily living), adequate nourishment and shelter. Some difference between facilities in the availability of staff to provide health and personal care were mentioned above concerning CHFC life.

Residents in unregistered facilities were more likely to have to retain the ability to self-care or to provide support to each other than those in registered facilities. Dev and other residents in the same facility noted:

*Here we don’t have anybody to get help. That is the rule here. One should eat oneself, one should wash oneself, one should sleep oneself. They are strict about it. Suppose you cannot walk by yourself, they, your co-residents may help you. That’s what happened to me for some 10 days. My roommates helped me. They would bring food for me.* (Male, 72 years, never married, H15 unregistered)

However, this was not common to all unregistered facilities. Although there were no ‘paid’ attendants, in one facility a nun helped residents with personal care tasks, as Deepak said:

*This Sister [name] takes me all by herself from the bed to the wheelchair. Takes me to the toilet for evacuation and cleans, gives me a bath, towels and brings me back here, dresses me and makes me lie down. She helps me eat food. She does everything in a good manner.* (Male, 68 years, widowed, H16 unregistered)

In the only unregistered facility with a care attendant (the manager) the ratio of care attendants to residents was 1:20, whereas in the registered facilities, it was around one person for every four or five residents. The difference in levels of staff between registered and unregistered facilities was particularly stark for the largest facilities: whereas the registered facility had 19 staff for 65 residents, the largest unregistered facility had only three staff (one manager and two cooks) for 110 residents. In this facility the manager explained that “*we give work to those who are able among the residents”*. Many of the manual jobs described by the manager, such as sweeping and cleaning rubbish are associated with lowest castes in India and are considered degrading [28].

*We assign the older among them such work as making brooms with coconut leaflets. If they are young, we assign cleaning and gardening work. But we rotate the tasks. For the mentally retarded elders [sic], I ask them to take the firewood... I will give the vegetables to them and ask them to handover to the cook… They do such things as sweeping and removing cobwebs. They clear the dustbins. We ask them to help their fellow residents who are bedridden.* (Manager, H11 unregistered)

In registered facilities, residents were more likely to receive support with personal care and medical or health care, even if this involved making clinical appointments outside the facility. In one facility some difficulties with personal support were noted: Pratik and Padma highlighted issues associated with assisting men and women to dress appropriately and with dignity.

*There is a lady nurse. She takes me to the bathroom and gives me a bath and helps me dress. But she is a woman, and she does not know how to tie the veshti. Other men around will come to help at such times.* (Male, 60 years, separated, H18 registered)

*That nurse gave me this petticoat without any saree. She is a nurse. Doesn’t she know that this petticoat is only suitable for a saree?* (Female, age unknown widowed, H18 registered)

To support the nourishment of residents, most facilities had a set weekly menu. Residents in most facilities were satisfied with both the quantity and quality of the food that they received and Hitendra’s comment was typical of many “*The food is good. Even at home we will not get such food”.* There were only two facilities in which residents indicated some dissatisfaction with the availability of food and drinks. In the first facility, this was mainly in relation to ‘snacks’ that had to be purchased. This was problematic for residents such as Varsha and Udit who had insufficient income.

*Here they make coffee occasionally. It is black coffee. We don’t get it daily. They give biscuits rarely. If we give money, we can ge*t. (Female, 75 years, widowed, H11 unregistered)

*I would like to eat some snacks like biscuits and omappodi. But I cannot get these.* (Male, 80 years, separated, H11 unregistered)

In the second facility (H18, registered), the quality and range of food provided did not suit Padma’s food preferences or intolerances, she said:

*Sour dosai. I don’t like it. If I eat this I will get leg pain. I don’t eat curd. I was advised not to eat sour things. They give just four idlies and they too will be sour. I will eat wheat dosai, but they will not give me any.* (Female, age unknown, widowed, H18 registered)

In terms of providing shelter the cleanliness of the unregistered facilities varied, and this is contrasted in the following quotes from Maalia and Hitendra. Whereas Maalia had to clean faeces from the bathroom before she bathed, Hitendra was very satisfied with the cleanliness of the old age home in which he lived.

*It [the bathroom] is befouled with urine and faeces. I clean it up with water and then, if I can tolerate it, I take a bath or wash clothes. I keep the clothes on my thigh and apply soap. What else can I do? Where can I go?* (Female, 75 years, widowed, H11 unregistered)

*The rooms and the beds are neat. They change the bed sheet every month. They sweep daily. Bathroom and toilet are clean.* (Male, 83 years, separated, H10 unregistered)

Bodily health is underpinned by opportunities to have good health (i.e. access to health and personal care), to be adequately nourished, and have adequate shelter. The Tamil Nadu Minimum Standards for old age homes specify the services that should be provided to residents. These include three meals (breakfast, lunch and dinner), two refreshment breaks (tea, coffee and snacks), and weekly visits by a medical officer. Furthermore, in-house staff should include a nurse, counsellor, cook and helpers (care attendants). While these services were more likely in registered homes there was still variability in terms of the quality of the services provided, an issue that is not addressed in the Minimum Standards. Overall, unregistered old age homes were less likely to provide opportunities for bodily health for residents: only one unregistered old age home in the study attempted to cater for the personal care needs of residents.

### Bodily integrity

Bodily integrity refers to moving freely from place to place, secure against assault. The themes ‘abuse’ and ‘leaving the premises’ (i.e. freedom to move within and beyond the old age facility to the community) were incorporated in this family node.

Residents in H14 (registered) and H10 (unregistered) were permitted to leave the premises if they gave written notice and were accompanied by an attendant. Special occasions such as weddings and birthdays often warranted longer trips away from the facilities, and Joti noted that residents could be accompanied by their relatives. Avinesh also mentioned that residents were permitted to go to local places if they were accompanied by a member of staff:

*If a resident wants to go out, like attending a wedding, the person who brought the resident here should come and take the resident.* (Female, 84 years, widowed, H14 registered)

*The reason is that we are all old and if anything happens it will become difficult. If we request and if it is a nearby place, they will send us with an attendant.* (Male, 78 years, married, H14 registered)

Only Dev mentioned being permitted to go out alone.

I *can go and come alone. They allow for it. But one should go and come back properly. If we do anything unwanted, they will not allow. When they have confidence in us, they allow.* (Male, 72 years, never married, H15 unregistered)

H11 (unregistered) particularly stood out in terms of denying residents freedom of movement. In this facility, most residents talked about their desire to leave and lamented the fact that they were not permitted to do so. Aanav’s reaction to a question about access to the local community was typical of residents in this facility, who expressed a desire to leave the old age home for good.

*I am only thinking of when to leave this place. Even if I have to beg for food… I want to go somewhere. I don’t want to be here*… *If you raise the walls and put a tiger alongside, we cannot escape. Now I am with that tiger [the manager] here.* (Male, 60 years, widowed, H11 unregistered)*.*

However, it was not only unregistered facilities that failed to support bodily integrity for residents. Padma noted that she was denied access to other areas of the old age home and said ‘*here we cannot move from one room to another’.* She also cited an example of abuse by staff when she was initially left at the home, deserted by her family and distressed:

*They first kept me on the staircase. As I kept on shouting, ‘Father Yesappa, save me!’ they took a plastic tea cup and gagged me. I fainted. Madam [the manager] went to her home. When I became conscious, I started chanting a prayer. The woman in the other room informed them. Madam came and ordered, ‘Don’t sing. Don’t pray. Shut up your mouth and lie down’.* (Female, age unknown, widowed, H18 registered)

Deprivation of freedom of movement was not only a feature of old age homes in Indian society. A summary of Maalia’s life history demonstrates how actions assumed to improve her life (and that of her daughter) diminished her freedom and subjected her to unequal relations (Female, 75 years, widowed, H11 unregistered). Maalia spent the majority of her life in various facilities run by the same charitable organisation. At a young age, Maalia admitted herself to a children’s home to avoid abuse at home. She left briefly to marry but was abandoned by her husband when she was six months pregnant. Maalia left her daughter in a children’s home, moved into a women’s refuge and worked in the kitchen of the orphanage that she had been raised in. She borrowed ₹3,000 from the organization to arrange her daughter’s marriage (despite the organisational commitment to find suitable grooms for female residents, *and* meet all of the associated costs), and later required ₹27,000 for hospital fees to treat a burn sustained while working in the kitchen. After the first ‘loan’, the proprietors retained her salary (₹500 per month) for more than two decades. Eventually, Maalia’s sight deteriorated and she needed eye surgery. Unable to work to pay back another loan, Maalia requested to move to an old age home for older people that was located within the cluster of facilities. Thus, Maalia’s experience in the old age home was the result of a cumulative sequence of events. Deprivation of freedom was coupled with coercion through indebtedness to the cluster of charitable facilities. She suggested that death was preferable, “*I want to pass away as soon as possible. I should hurry to vacate this place*.”

With the exception H11 (unregistered), most residents were permitted to leave facilities if they were accompanied by a relative or care assistant. However, access to the community was not equal among residents. Padma (H10, registered) noted that she was not permitted to move around the facility, or to leave, whereas other residents in the same facility were able to go out if they were accompanied. Across all facilities, residents who were unable to walk (e.g. confined to bed) were rarely provided with sufficient support to move around the facility, and were not given sufficient support to leave the facility (see section on play). The Tamil Nadu Minimum Standards for old age homes have given scant attention to this particular facet of dignity for residents. The only reference to leaving the facility is in relation to ‘outings’ in which it is stated that “*The inmates should be taken out on local outings like temple, fairs, plays and places of tourist interests at least once in 3 months”*. This suggests that old age homes should offer planned activities, rather than facilitating the freedom of movement for residents.

### Play

Securing dignity through play, concerns providing residents with the opportunity to laugh and enjoy recreational activities. Several old age homes provided residents with newspapers, books and opportunities to watch the television. One old age home (H14, registered) which provided accommodation and care for Brahmins, appeared to have the most ‘occupied’ residents. This facility provided residents with a range of religiously oriented activities such as chanting mantras, prayers, reading spiritual books, watching religious series on television, and singing devotional songs. On the other hand, residents of H10 (unregistered) were mainly reliant on the television and newspapers for recreational activities, as Hitendra noted:

*We will get newspapers at 10 am. We get four newspapers... We also get magazines... Back at home, we had to walk some distance to go to a library… They put the TV on by 9.30 am, but I don’t have the habit of watching TV. I have to read all the four newspapers.* (Male, 83, separated, H10 unregistered)

There was evidence that some old age homes (H11 unregistered and H18 registered) did not provide any leisure or recreational activities. Instead, in H11 the residents who were able to work were given jobs. For example, Varsha (Female, 75 years, widowed) said “*I sit at that gate [entrance of the home] and my work is to open and close it”*. Saksham (Male, 84 years, widowed) said that residents who were unable to work were “*Sitting quietly… Nothing else”*. Despite paying fees, there were no leisure activities for residents in H18, and Pratik noted:

*Breakfast will be over by 9.30 am. Then I just sit. At 1 o’clock there is lunch. From 1.30 to 4 pm, we get time to recline. What else do we need at this old age? But we have to pay for all these.* (Male, 60+ years, separated, H18 registered)

One old age home deliberately denied residents the opportunities for recreation, as described by Rajiv:

*There is a TV in that hall. If we go there to watch it, they will switch off and say that there is no power supply, but if we come back to the room and put on the fan, it will work. So, they don’t like us to watch TV… Sometimes we get parcel food that is wrapped with old newspapers. I would carefully unwrap it and keep it for reading. I used to read the same paper again and again. You know, what they would do? They would select the food parcel with dampened wrapper and give it to me so that I cannot read it*. (Male, 63 years, never married, H16 unregistered)

In other registered and unregistered old age homes, access to leisure activities was inequitable for certain residents. For example, there were few opportunities to participate in recreational activities for residents who were nonambulatory or tetraplegic, such as Deepak and Rina.

*I can read newspapers. But there is no one to hold the newspaper for me. So, I don’t have anything else to do. It is just sitting or lying. If I am seated, I would keep on sitting until somebody comes and puts me to bed.* (Male, 68 years, widowed, H16 unregistered)

*I cannot get up. I cannot sit… My only problem is that I don’t have anybody else here to talk to. I am always lying down. If they put on the TV, I will listen to the news. I don’t go to the hall and watch TV. Who will take me there?* (Female, age unknown, never married, H18 registered)

The narratives indicated considerable variation in the extent to which residents in old age homes are supported to ‘play’. Whereas some homes met the Tamil Nadu Minimum Standards which stated that *“games should be played in the evening singing songs (devotional) and other past time activities may be designed depending on the age category and health status of the inmates”* and that recreational facilities (e.g. books, indoor games, radio, and television) should be made available, others failed to provide any facilities, or denied residents access to these.

## Discussion

Registration of old age homes is mandatory in Tamil Nadu. However, many remain unregistered. To date, Minimum Standards are only enforced in homes that are registered and receiving funding, as these are the only homes that the State is aware of. The results show that there is considerable variation in the extent to which the four CHFC life, bodily integrity, bodily health and play are met for older people living in these facilities. Furthermore, variation is not necessarily between old age homes that are registered versus those that unregistered. In essence, there is evidence that Articles 3, 13, 25 and 24 of the UDHR are contravened in both registered and unregistered old age homes in India. This suggests that the State (the Government of India) is not meeting its obligations under Article 1 to recognize that *‘all human beings are born free and equal in dignity and rights’* and has failed to mandate and implement safeguards for all older residents. In registered homes, it appears that Standards are not being regulated through inspection, nor is support offered to help maintain quality where old age homes fall short.

Considering the CHFC ‘life’ and Article 25 of the UDHR, in long-term care facilities and other healthcare settings around the world, routine clinical decisions are made about whether to treat older people at the end of life, or to prevent a life from being prolonged. The idea ‘that a life is not worth living’ is used to support these decisions [29]. However, in this study, in one unregistered home untrained, non-clinical staff (e.g. members of a religious order) were making judgements and withholding both curative and palliative care to residents. Furthermore, in four of the ten facilities, there were no health or support staff to secure appropriate and timely health care for residents, to ensure that they did not die prematurely.

A majority of older people requiring health care and support at the end of life in India – either living in the community or in old age homes—do not have access to services [30]. This is reflected in India’s poor global ranking on the 2015 Quality of Death Index, that places it 67^th^ out of 80 countries [31]. In 2014, the World Health Assembly passed a resolution to strengthen palliative care as a component of comprehensive care throughout the life course and urged national governments to carry out actions to develop palliative care (WHA67.19). In this respect, the education, clinical training, and competence of staff in old age homes are pre-requisites to facilitate dignity [24]. The State needs to ensure that old age homes are adequately staffed to secure health care for residents, and that staff are sufficiently skilled to uphold the rights of residents to a good life (and death).

Turning to bodily health and the associated Article 25 (emphasising access to health care and personal support, food and shelter as the foundations of health, wellbeing and a dignified life), results indicated considerable variation between old age homes. In some facilities human rights were violated, with residents living in filthy conditions, while others were expected to help each other without any other provision for personal care or support within the facility. Elsewhere in the world, studies identifying risk factors for neglect have found that staff shortages, time pressures, staff turnover, and a high ratio of residents to staff contribute to care quality [32, 33]. Thus, the dignity of care and support afforded to residents in some old age homes in India, suggests that the State needs to develop policies and strategies that regulate staffing ratios but also attend to quality of care and the maintenance of dignity.

Considering bodily integrity and Article 13: the right to freedom of movement, the study showed that most of the old age homes considered the safety of the residents and permitted them to leave accompanied by relatives or staff. However, residents are described as ‘inmates’ in policies (e.g. the Tamil Nadu Minimum Standards is published in English), programmes, and in research publications on old age homes emanating from India [34, 35]. This is the language of incarceration. The term ‘inmates’ has been rejected for prisoners as it is derogatory, stigmatising, and dehumanising [36]. We contend that it is inappropriate to use ‘inmates’ to describe old age home residents for these same reasons, but also because it reinforces the notion that imprisonment, deprivation of liberty and segregation from the community is legitimate. While the deprivation of liberty of old age home residents is governed by legal codes in most European Countries [37], the decision to detain residents in India is arbitrary. The results showed that some residents were detained against their will, violating their human rights and undermining their capacity to live a dignified life.

The results of the study indicated that both bodily integrity (freedom of movement) and play are more frequently overlooked when residents have higher level needs, for example, are nonambulatory. Under these circumstances, some residents were denied their human rights with fewer (if any) opportunities to leave the premises or to engage in recreation. Elsewhere, studies have indicated that many care home residents spend a large proportion of the day inactive [38]. This is particularly salient for residents with dementia where there is evidence of restrictive practices, confinement and systematic breaches of human rights in care homes [39]. Severe physical or cognitive impairment is likely to incur greater demands on staff time to support freedom of movement and opportunities for leisure, when compared to the level of support required by residents who are less impaired. However, based on the concept of status dignity, it is the duty of the State (and its agents in old age homes) to uphold the human rights of all older people even if this requires additional staffing to ensure equity in securing CHFC for residents.

### Limitations

The study was conducted in only one state in India, Tamil Nadu, and there may be variation in the quality and types of support provided in old age homes across India. However, we have no reason to believe that we would find a higher ‘standards’ of provision elsewhere. In 2019, the Ministry of Housing and Urban Affairs, Government of India developed a set of ‘model guidelines’ that are applicable to real estate developments intended for older residents who are ‘*willing and able to pay for accommodation services and facilities’* [40]. These model guidelines focus on services and physical aspects of the environment rather than the quality of care. The authors are fairly confident that the types of human rights violations observed in this study, would be found elsewhere in India (see also, [41]). As Tamil Nadu was the first state to introduce a set of Minimum Standards for old age homes in 2016, one may expect provision in this state to be ‘better’ than elsewhere as the standards have become embedded into practice over time.

This study was undertaken before the COVID-19 pandemic was declared a Public Health Emergency of International Concern by the World Health Organization in 2019. Globally, the pandemic has resulted in human rights violations for older people, especially in relation to the right to health and life [42]. Policy directives that were developed to protect the life of residents in care homes, have also impacted on bodily integrity and play [43]. Therefore, the experiences of residents in old age homes in Tamil Nadu are unlikely to have improved over the last two years. As old age homes are largely unregulated it is unlikely that the full extent of the impact of the COVID-19 pandemic on the human rights of older residents in India will be established [44].

## Conclusions

Residents in old age homes can function effectively in the range of areas that are fundamental to a fully dignified human when policy decisions and the legal apparatus of the State provide them with the opportunities to do so. The concepts of status dignity, CHFC, and human rights have been used to describe the relationship of residents in old age homes to the State and the agents of the State (staff in old age homes). The results suggest that a new legislative framework is required to ensure that all old age homes are accountable to the State, regardless of the source of funding. We recommend that Minimum Standards include clear definitions regarding the expectations for quality of care and dignity in care, that meet the basic needs of older people (shelter, clothing and food) but also provide health care, personal support, and opportunities for leisure, socializing and access to the community. The legislative framework should also stipulate staff ratios, staff training and raising awareness of human rights. Standards should be regulated and support offered to help maintain quality. The study has highlighted incidents where human rights have been violated, but these illustrative examples have been juxtaposed with good practice, where residents’ human rights and dignity were protected. The research has demonstrated that it is possible to protect the dignity of residents of old age homes, but highlights areas where the Government of India and/or State Governments have a role to play in strengthening and developing old age home policies and strategies to protect older residents.

## Data Availability

The datasets generated and/or analysed during the current study are not publicly available as restrictions apply to the availability of these data (intention of data analysis included in participant information forms) and sensitivity (i.e. human data) but are available from the corresponding author on reasonable request. Data are located in a controlled access repository at the University of Auckland.

## Abbreviations

CHFC: Central Human Functional Capabilities
UDHR: Universal Declaration of Human Rights
